# Proceedings of the Rock River Union Medical Society

**Published:** 1859-08

**Authors:** G. W. Phillips

**Affiliations:** Secretary


### MISCELLANEOUS MEDICAL INTELLIGENCE.

### PROCEEDINGS OF THE ROCK RIVER UNION MEDICAL SOCIETY.

The annual meeting of the Society was held at Sterling, June 15th.

The President, Dr. J. A. Jackson, being absent, Dr. D. Williams, of Sterling, was chosen President *pro tem.*

Proceedings of last meeting read and approved. Constitution read.

On motion, Dr. Wm. J. Galt of Sterling was admitted a member of the Society.

Dr. G. W. Phillips, chairman of the Committee on Practical Medicine, made a report, which, on motion, was adopted, and the Secretary directed to place a copy of it on file.

A discussion arose in regard to that portion of the report, in relation to the "Effects of Alcoholic drinks in Tuberculosis," or upon those predisposed to the disease.

The discussion was participated in by Drs. Abbott, Hudson and Phillips.

A case of Rabies having recently occurred at Dixon, was reported by Dr. Abbott. The patient, an Irishman; occupation, well-digger; age about fifty. He was bitten in the hand by a rabid dog six weeks before the disease manifested itself. Symptoms: the attack commenced with chilly sensations, and confusion of intellect, soon followed by slight spasms of the muscles of respiration and deglutition upon attempting to swallow food or drink; a constant sense of constriction about the throat and chest; respiration but little affected; pulse rather weak and contracted; occasionally would swallow water by bringing the cup up behind him, so as not to see it; third, vomiting and spitting of viscid mucus; mind clear, but irritable; he showed no disposition to injure any one; the bitten limb was painful, and was partially paralyzed; the least breath of air was annoying to him; general convulsions did not occur; he died while sitting in his chair, thirty-six hours from attack.

He was seen by the medical fraternity of Dixon, but refused all treatment except what was given him by a root doctor.

Drs. Royer and Hudson stated, that there had been three cases of persons bitten by mad dogs, at Sterling, within a few days. The treatment made use of, was excision of bitten part, and application of caustic.

Dr. Hudson was appointed to make a report upon—"The curative effects of opium in febrile and inflammatory diseases," said, he had prepared a paper, but not having a copy, gave a synopsis of the report.

Dr. Phillips reported a case of mortification of the lungs, occurring as a primary disease, not being preceded by a single symptom common to acute inflammation.

The Secretary directed to place a copy on file.

On motion of Dr. Phillips, a committee of three were appointed, consisting of Drs. Donaldson, Phillips, and Royer, to revise the present fee-bill, and report at next meeting.

Dr. Donaldson introduced the subject of scabies, the pathology and treatment of which was discussed by Drs. Donaldson, Abbott and Phillips.

The officers elected for the ensuing year, were:—For President, Dr. H. C. Donaldson, of Como; Vice-President, Dr. Moses M. Royer, of Sterling; Secretary, Dr. G. W. Phillips, of Dixon; Treasurer, Dr. H. S. Hudson, of Sterling.

On motion of Dr. Hudson, the President and Secretary were authorized to appoint delegates to the National Medical Association and State Medical Society.

On motion of Dr. Phillips, Dr. A. S. Hudson was appointed to deliver the popular address at next meeting.

On motion of Dr. Hudson, Dixon was selected as the place for holding the next semi-annual meeting.

Dr. J. B. Porter, who was appointed to deliver the popular address at this meeting, being absent, no address was forthcoming.

On motion of Dr. Hudson, the Secretary was directed to prepare a synopsis of the proceedings of the meeting, and send it for publication in *Chicago Medical Journal.*

On motion, adjourned.

G. W. PHILLIPS, *Secretary.*

